# Interferon-induced transmembrane protein 3 in hepatocellular carcinoma patients

**DOI:** 10.1186/s12885-023-11071-2

**Published:** 2023-06-23

**Authors:** Rania M. Bondok, Lamiaa A. Barakat, Alyaa R. Elsergany, Nancy Mahsoub, Maivel H. Ghattas

**Affiliations:** 1 Department of Biochemistry, Faculty of Science, Port-Said University, Port Said, Egypt; 2 Internal Medicine Department, Oncology Center, Faculty of Medicine, Mansoura University, El Mansoura, Egypt; 3 Department of Clinical Pathology, Faculty of Medicine, Mansoura University, El Mansoura, Egypt; 4 Department of Medical Biochemistry, Faculty of Medicine, Port-Said University, Port Said, Egypt

**Keywords:** Interferon-Induced transmembrane protein 3, Hepatocellular carcinoma, Hepatitis C virus, Gene polymorphism

## Abstract

**Objective:**

The study aimed to investigate the over expression of IFITM3 in hepatocellular carcinoma Egyptian patients.

**Background:**

Hepatocellular carcinoma (HCC) continues to be a serious disease burden. Interferon Induced Transmembrane protein 3 (IFITM3) is a protein that encoded in humans by the IFITM3 gene. It plays a critical role in the immune system’s defense, responsible for a large portion of the antiviral activity. In this study, we showed that IFITM3 rs 12252-CC was over expressed in HCC patients compared to control group with HCV infection.

**Method:**

DNA sequencing was applied for detection of IFITM3 rs 12252-CC and IFITM3 protein level was measured by ELISA to 50 patients with HCC with cirrhosis and 50 with Hepatitis C virus infection.

**Results:**

The obtained results of this study indicated that IFITM3 rs 12252-CC was significantly elevated in HCC group, the codominant model of CC genotype of IFITM3 gene had high association with risk of hepatocellular carcinoma with odd ratio (OR) = 2.70, p = 0.041.

**Conclusion:**

IFITM3 play an important role in progression of hepatocellular carcinoma. Results revealed that IFITM3 rs 12252-CC among Hepatocellular carcinoma patients would allow diagnosis and starting intervention.

**Supplementary Information:**

The online version contains supplementary material available at 10.1186/s12885-023-11071-2.

## Introduction

Hepatocellular carcinoma is the most common type of primary liver cancer, accounting for 85–90% of all cases. Importantly, only a small percentage of HCC patients are detected at an early stage, when curative treatments are most effective [1].

Hepatocellular carcinoma is a typical slowly developing malignancy mainly stimulated in the form of chronic inflammation from exposure to infectious agents or harmful substances. The aggregation in the tumorigenic process different neoantigens are formed by genomic alterations, which can induce immune responses [2].

The proteins in IFITMs include a CD225 domain composed of transmembrane domain 1(TM1), a cytoplasmic domain, and two exons that encode transmembrane polypeptides. The common domains include N- and C-termini, two transmembrane domains, and a conserved cytoplasmic domain. The N-terminal portion of IFITM3 is one of these domains, and it is essential for the protein’s optimal cellular localization. If the N-terminal 21 amino acids are eliminated, IFITM3 loses its connection with the endosomal compartments and migrate to the cell surface, eliminating its antiviral activity [3].

In late endosomes exists IFITM3, there are numerous IFITM3 single nucleotide polymorphisms (SNPs) in the translated sequence of IFITM3, and the rs12552 CC genotype has been correlated to hepatocellular carcinoma with poor differentiation, high development, and a greater relapse rate [3].

The present study aimed to elucidate the association between the IFITM 3 rs 12252 gene polymorphism in Egyptian patients with hepatocellular carcinoma (HCC) and the control group (HCV) and to reveal the genotype in C virus patients considering C virus infection is one of the most common causes of HCC. and study the relation between the genotypic frequencies for these gene and the biochemical measurements, hematological parameters and the tumor marker Alpha Feto Protein (AFP). And to achieve our purpose, we determined level of IFITM-3 protein and the relation to genotypes of IFITM 3 rs12252 and routine parameters..

## Methods

### Patients

This study included 100 patients would be classified into two groups for comparison, 50 patients they were diagnosed hepatocellular carcinoma their age average (40–75) years, they were 39 males and 11 females, and 50 patients with HCV their age average (33–65) years, they were 23 males and 27 females without HCC (The control group were chosen from patients with positive HCV which was with normal levels of AFP and triphasic Computed Tomography were free from any mass to exclude possibility of having HCC). Patients were collected randomly from El-Mansoura oncology center during the interval between November 2019 and August 2020.

### Collection and handling of samples

Sample of venous blood from patients was collected on Ethylene di amine tetra acetic acid (EDTA) tubes for analysis of gene polymorphisms of IFITM3 rs 12252-CC and hematological measurements (Hemoglobin, platelets and white blood cells), another sample was collected and centrifuged for 20 min at the speed of 2000–3000 rpm, then separation of serum which was transferred into another tube and kept frozen at -20 °C for assessment of IFITM3 protein and biochemical tests. Routine laboratory investigations including kidney functions (Uric acid and Creatinine), liver functions (ALT, AST, Total bilirubin and Albumin), α-fetoprotein tumor marker (AFP) and viral marker (HCV antibody)..

- Determination of the IFITM3 protein level in human serum was determined by using ELISA technique.

### Genotyping assay of IFITM3 by DNA sequencing analysis

DNA purified from whole blood using the DNA extraction kit QIAamp DNA Blood Mini Kit #51,104.

The human IFITM3 rs12252 was sequencing by polymerase chain reaction (PCR). The amplification performed using the following forward and reverse primers.

(F-primer) 5’GGAAACTGTTGAGAAACCGAA 3’ and.

(R-primer) 5’ CATACGCACCTTCACGGAGT 3’.

The PCR products were purified and sequenced then single nucleotide polymorphism was identified using sequencing capillary 310 system. Samples were subjected to direct DNA sequencing to validate the genotyping result. After PCR purified DNA fragments sequenced, the DNA sequence compared to the corresponding sequence of the IFITM3 rs12252 gene.

### Statistical analysis

Statistical analyses performed using SPSS Version 22.0 and graph prism 8. All continuous variables in this research were non-normally-distributed. Variables reported as medians. Non-parametric tests used to compare continuous variables. Receiver operating characteristic (ROC) curves, area under the curve (AUC), Correlations between SNP and severity were assessed using adjusted odds ratios (OR) and 95% CI were calculated using odd ratio calculator. The Hardy-Weinberg equilibrium calculated through the http://oege.org/software/hwe-mr-calc.shtmlwebsite, as well as OR and 95% CI, which were adjusted. P-values (A two-tailed value of P < 0:05 was considered to indicate a statistically significant result).

## Results

Before analyzing IFITM3 expression in hepatocellular carcinoma patients, we noted the distinguishing factors for the patients as listed in Table 1 the age of the cancer patients ranged from 40 to 75 years, the proportion was male (78%) and female (22%), and the patients with cancer had a family history (52%). The percent of diabetics (36%), Hypertension patients (30%) and smokers (34%). Patients with a tumor mass greater than 3 cm (68%) and a tumor mass less than 3 cm (32%), splenomegaly with B symptoms (56%), child class A patients (18%), child class B patients (44%). Hepatocellular carcinoma (HCC) patients with distal metastasis (26%) were included. In this study, there were 23 (46%) males and 27 (54%) females in the control group of Hepatitis C patients between the ages of 33 and 65.

**Table 1:** Patient’s Characteristics of the HCC group

variables	Cancer patients’ numbers (% of group)
**Age** median (max-min)	61(75-40)
**Sex** Male Female	39 (78%) 11 (22%)
**Family history**	26 (52%)
**Diabetic**	18 (36%)
**Hypertension**	15 (30%)
**Smoker**	17 (34%)
**Tumor mass size** **≥** 3 cm **<** 3 cm	34 (68%) 16 (32%)
**Splenomegaly** Present (with B Symptoms) Absent	28 (56%) 22 (44%)
**Child Score** A B	9 (18%) 22 (44%)
**Distal metastasis** Present Absent	13 (26%) 18 (36%)

**Fig. 1:**
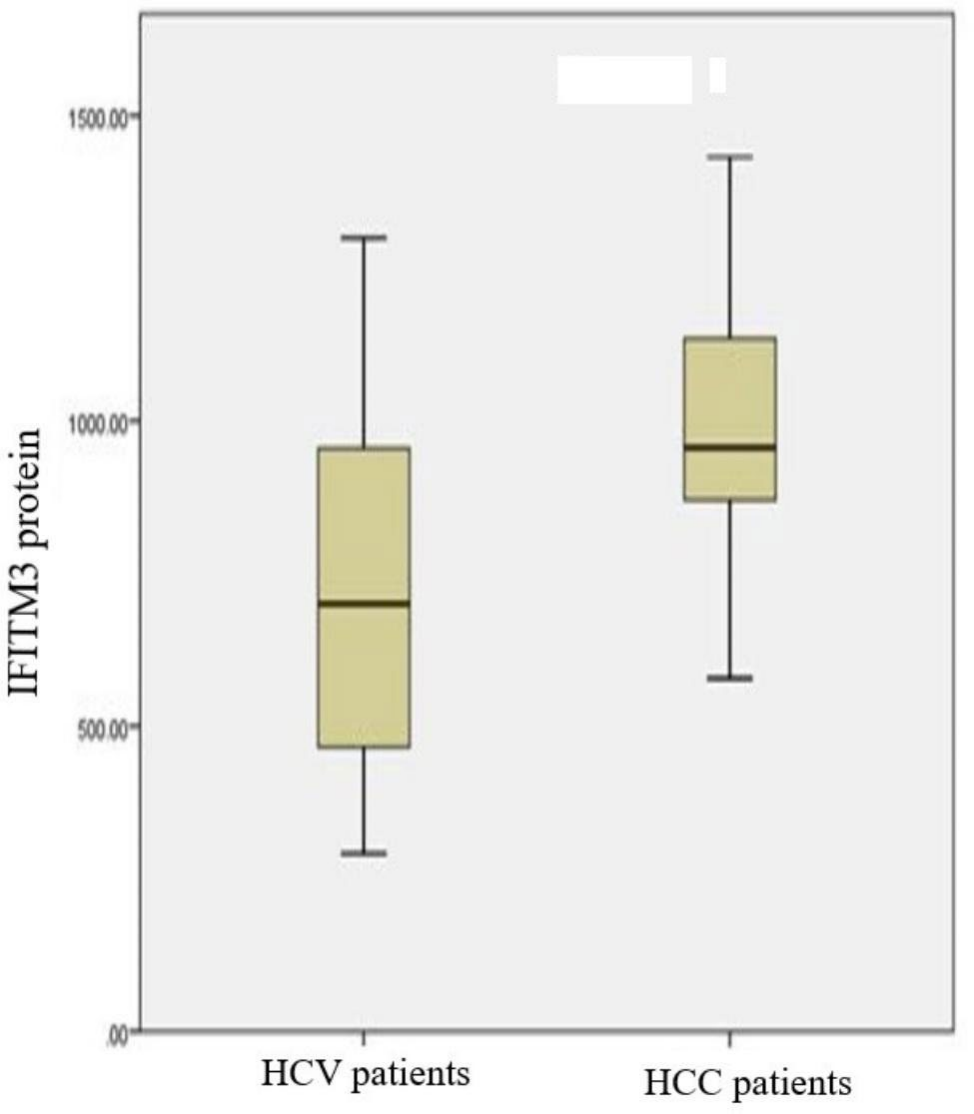
Boxplot of serum IFITM3 protein in cancer patients compared to control group. The box represents the interquartile range. The whiskers indicate the highest and lowest values, and the line across the box indicates the median value

There was no significant difference between cancer patients compared to control group in biochemical parameters albumin and uric acid but it was clear that there is a significant difference in biochemical parameters Alanine aminotransferase (ALT), Aspartate Aminotransferase (AST), Total Bilirubin, (p = 0.001) and Creatinine, (p = 0.03). There was a significant difference with tumor marker Alpha Feto Protein, (p = 0.001). There were no significant differences in the hematological measurement platelets, (p = 0.47) but there was a significant difference in WBCs, (p = 0.02) and Hemoglobin, (p = 0.01). The protein marker IFITM3 indicated a significant difference between cancer patients and control group, p = 0.004 as shown in Table 2. The protein level differs in cancer patients than control group, Fig. 1 shows high level of IFITM3 protein in cancer patients.

**Table 2:** The parameters of cancer patients compared with control group

parameters	Cancer patients (N = 50)	Control group (N = 50)	p
	Median(max-min)	Median(max-min)	
ALT (U/L)	37(56 − 25)	36(57 − 19)	0.001^*^
AST (U/L)	38(60 − 20)	52(75 − 33)	0.001^*^
Total bilirubin (mg/l)	0.9(1.9 − 0.4)	0.6(1.3–0.3)	0.001^*^
Albumin (g/l)	3.5(4.8–1.98)	3.6(4.9–2.6)	0.78
Creatinine (mg/l)	0.9(1.4–0.4)	0.8(1.4 − 0.1)	0.032^*^
Uric acid (mg/l)	5.2(8.5–2.3)	4.9(7.6–2.7)	0.214
AFP (ng/ml)	162.5(210 − 87)	3(5.4–1.1)	0.001^*^
WBC (x10^3 / µL)	7.05(12 − 1.6)	6.9(10.6–1.9)	0.025^*^
Hemoglobin (g/l)	12.5(15.4–7.5)	11.6(14.6–5.2)	0.017^*^
Platelets (x10^3 / µL)	218(425 − 35)	191.5(286-16.78)	0.47
IFITM3 (ng/dl)	956(1369 − 547)	700(1299 − 291)	0.004^*^

Data are presented as median (range)

* Statistically significant compared with control at P < 0.05

### Correlation analysis

Correlation analysis was determined among some parameters and the protein level, our study showed that IFITM3 was positively correlated with AFP (r = 0.438, p = 0.001), Creatinine (r = 0.287, p = 0.004), Hemoglobin (r = 0.392, p = 0.001) and platelets (r = 0.300, p = 0.002). IFITM3 protein level was positively correlated with genotypic of IFITM3 (r = 0.373, p = 0.001) as shown in Table 3.

**Table 3:** Correlation between IFITM3 and parameters in cancer patients

Correlation of parameters		IFITM3 protein
AFP	r	**0.438**
	P	0.001^*^
Creatinine	r	**0.287**
	P	0.004^*^
Hemoglobin	r	**0.392**
	P	0.001^*^
Platelets	r	**0.300**
	P	0.002^*^
genotypic of IFITM3	r	**0.373**
	p	0.001^*^

* Statistically significant compared with control at P < 0.05

**Fig. 2:**
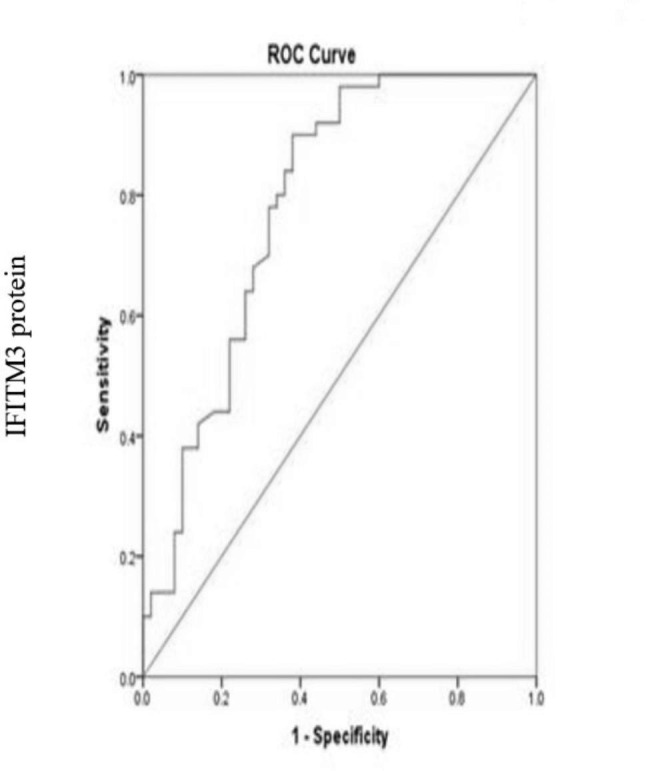
Receiver operating characteristics curve (ROC) of IFITM3 protein marker in hepatocellular carcinoma patients

Differences in biomarker levels between variables were calculated using ROC curve as shown in Fig. 2; we assessed the diagnostic of serum utility of IFITM3. In hepatocellular carcinoma patients, the best chosen cut off of serum IFITM3 was 835.5 ng/l yield AUC = 0.79, with a sensitivity 98% and a specificity 60% as shown in Table 4.

**Table 4:** The cut off level of IFITM3 in cancer patients

Result of Variable	Cut off	Area	Sensitivity	Specificity
IFITM3	835.5	0.79	98%	60%

### The frequencies of Genotypic for IFITM3 rs12252-CC

**Fig. 3:**
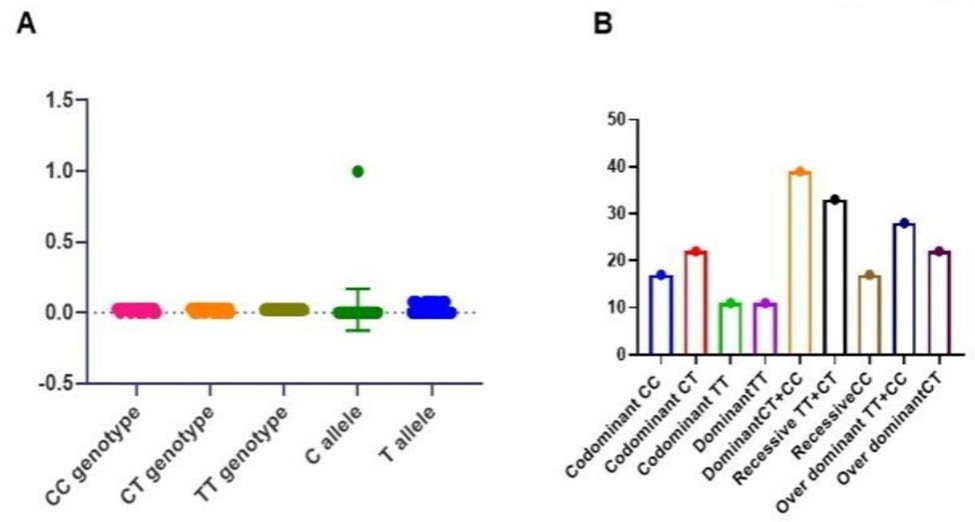
**(A)** The frequency of genotypes and alleles **(B)** models of the IFITM3 in cancer patients

For IFITM3 rs 12252-CC gene polymorphisms, the results in this study as showed in Fig. 3A the homozygous TT genotypic frequency in hepatocellular carcinoma patients (11%) that was less than which founded in control group (23%). For genotypic frequency of heterozygous CT, which found to be higher in cancer patients (22%) compared to control group (19%), the frequency of homozygous genotype CC in cancer patients (17%), while in control group was (8%).

For the allelic frequencies of C allele, there are statistically high significant differences between the cancer patients (n = 56) compared to control group (n = 35) where, p = 0.0031. Hardy-Weinberg equilibrium (HWE) showed that there were no significant differences comparison of the allelic frequencies in hepatocellular carcinoma and control group allele as shown in Table 5.

**Table 5:** The genotypic frequencies of IFITM3 gene polymorphism for hepatocellular carcinoma compared with control group

Genetic polymorphism of IFITM3	Cancer patients	control	OR (95% CI)	P
Genotypic frequency	n (%) 100	n (%)100		
CC	17	8	2.704 (1.039,7.035)	0.041^*^
CT	22	19	1.282(0.5768, 2.8494)	0.542
TT	11	23	0.380 (0.161, 0.897)	0.027^*^
HWE	X2 = 0.579 P = 0.749	X2 = 1.363 P = 0.506		
Allelic frequency	n (%) 100	n (%) 100		
T	44	65	0.423(0.239,0.748)	0.0031^*^
C	56	35		

* Statistically significant compared with control at P < 0.05

The codominant model of CC genotype of IFITM3 gene had high association with risk of hepatocellular carcinoma with odd ratio (OR) 2.70, p = 0.041, the codominant model CT genotype of IFITM3 gene odd ratio with hepatocellular carcinoma (OR) 1.282, p = 0.54 and the codominant TT odd ratio 0.38, p = 0.02 as shown in Table 5.

Figure 3B shows the models of IFITM3, the dominant model (CT + CC) of IFITM3 gene. There was an association of the risk of hepatocellular carcinoma with odd ratio 3.02, p = 0.01. The (TT) dominant model of IFITM3 had odd ratio 0.33, the recessive model (TT + CT) of IFITM3 had no association with the risk of (OR) 0.36, p = 0.04, the CC recessive model odd ratio 2.70 with high association with risk of hepatocellular carcinoma. In this study the over dominant model TT + CC for IFITM3 gene had no association to risk of hepatocellular carcinoma with odd ratio 0.78, p = 0.54, the over dominant model CT association to risk of hepatocellular carcinoma (OR) 1.28 as shown in Table 6.

**Table 6:** The distribution of genotypes and models of IFITM3 for hepatocellular carcinoma compared with control group

Model	Genotypes Of IFITM3	Cancer patients N (50)	Control Group (50)	Odd Ratio (OR)	95% Confidence Interval (CI)	P
codominant	CC CT TT	17 22 11	8 19 23	2.704 1.282 0.380	(1.039,7.035) (0.5768, 2.849) (0.161, 0.897)	0.04^*^ 0.54 0.02^*^
Dominant	TT CT + CC	11 39	23 27	0.33 3.02	(0.1387, 0.790) (1.265, 7.209)	0.01^*^
Recessive	TT + CT CC	33 17	42 8	0.369 2.70	(0.1421, 0.96) (1.039, 7.035)	0.041^*^
Over dominant	TT + CC CT	28 22	31 19	0.780 1.28	(0.3510, 1.733) (0.5768, 2.849)	0.54

* Statistically significant compared with control at P < 0.05

**Fig. 4:**
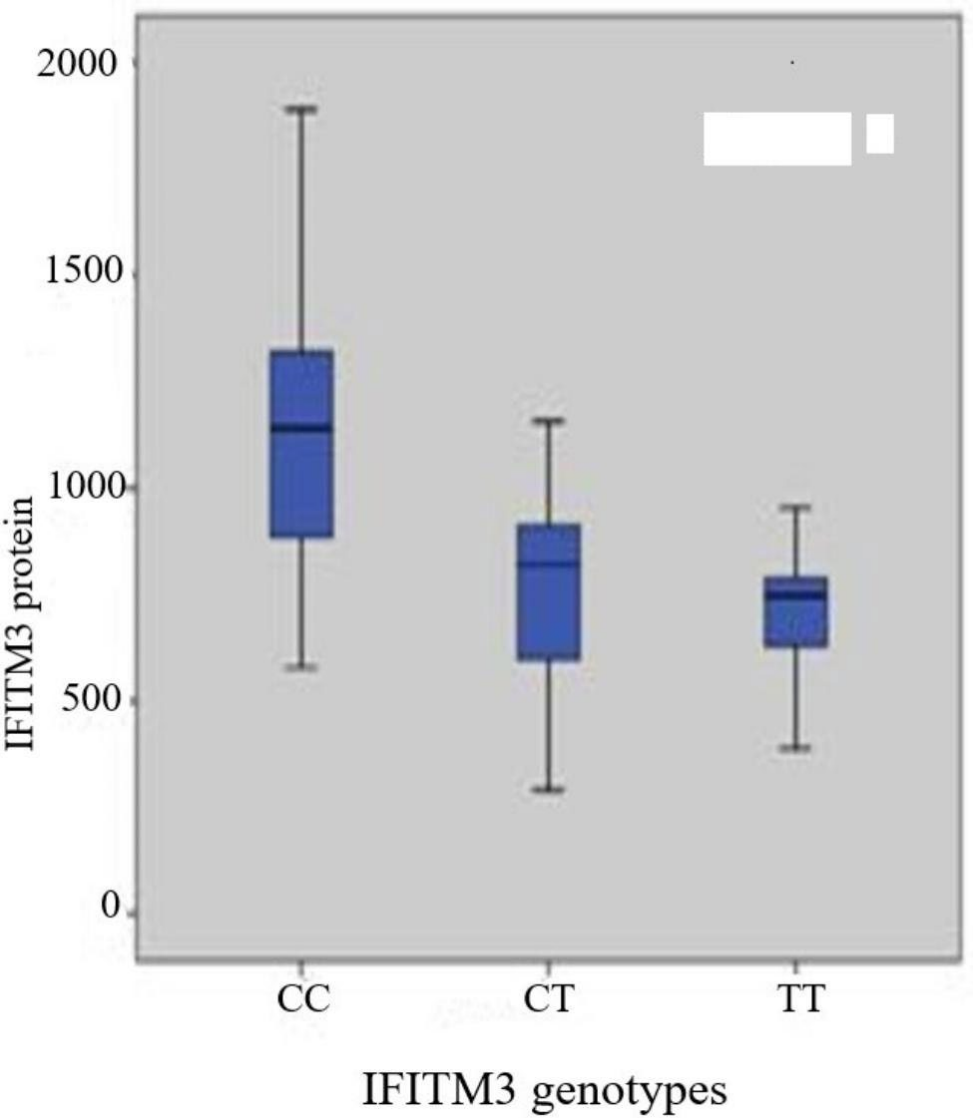
Boxplot of serum IFITM3 protein and genotypes of IFITM3 rs 12252-CC The box represents the interquartile range. The whiskers indicate the highest and lowest values, and the line across the box indicates the median value

Figure 4 shows a higher level of IFITM3 protein in the CC genotype than in the CT and TT genotypes.

Table 7. Showed that there were a significant between chemical parameters (Albumin, p = 0.008 and AFP, p = 0.001) in CC genotype of IFITM3. In CT genotype, there was a significant between chemical parameters (ALT, p = 0.05, Albumin, p = 0.001, Total bilirubin, p = 0.001 and AFP, p = 0.001), in genotype TT, there was a significant between Total bilirubin, p = 0.003 and AFP, (p = 0.001). IFITM3 protein showed a significant difference between CC, CT and TT genotypes (p = 0.01,0.01and 0.08) respectively.

**Table 7:** Biochemical parameters, IFITM3 protein and genotypic of IFITM3

Biochemical parameters	The genotypic of patients		
IFITM3	CC (n = 14)	CT (n = 51)	TT (n = 35)
ALT(U/L)	0.52	0.05^*^	0.39
AST(U/L)	0.98	0.16	0.63
Albumin(g/l)	0.008^*^	0.001^*^	0.23
Total bilirubin (mg/dl)	0.14	0.001^*^	0.003^*^
Creatinine (mg/dl)	0.23	0.71	0.56
Uric acid	0.47	0.50	0.18
AFP (ng/ml)	0.001^*^	0.001^*^	0.001^*^
WBCs	0.95	0.17	0.38
Hemoglobin	0.86	0.27	0.61
Platelets IFITM3 protein	0.16 0.01^*^	0.62 0.01^*^	0.99 0.08^*^

* Statistically significant compared at P < 0.05

## Disscussion

In terms of histology, genetic aberration, and protein expression, HCC represents a varied group of tumors [4]. HCC, one of the five most prevalent cancers, is a widespread liver tumor that is most frequently found in individuals with cirrhosis or chronic liver disease. HCC is additionally the most common liver tumor [5]. HCC is an epithelial tumor with liver origins that is composed of cells with features resembling those of normal hepatocytes [6].

About 80% of people infected with the hepatitis virus suffer from chronic infection that continues throughout their lives, and this percentage may develop serious liver diseases such as fibrosis, cirrhosis, and in some cases hepatocellular carcinoma [7].

Interferon-induced transmembrane protein 3 (IFITM3) is a double transmembrane protein with a molecular weight of 10–15 kDa. IFITM3 is known to be involved in a variety of biological processes, including as bone mineralization, immune-cell regulation, germ cell homing and maturation, and cell adhesion. A rising number of studies have found that IFITM3 is improperly expressed in a wide range of human malignancies and is likely involved in tumor progression. Metastasis is the most frequent cause of mortality in patients with HCC who have had a curative resection. The IFITM3 polymorphism rs12252-C encodes an isoform that functions differently from its normal type as it lacks 21 amino acids from the amino terminus. HCC progression and low differentiation is related to the rs12252-CC genotype [8].

A balance between high and low IFITM3 levels might be observed in the frequency of circulating genotype single nucleotide polymorphism (SNP) in the human population. rs12252 a minor IFITM3 allele, T > C, is a single nucleotide polymorphism [9].

This study, identified the role of IFITM3 rs 12252-CC and confirmed the results by indicated the levels of IFITM3 protein in serum. The results of this study supported that IFITM3 are significantly higher in HCC.

For IFITM3 rs 12252-CC gene polymorphisms, the results in this study showed that the homozygous TT genotypic frequency in hepatocellular carcinoma patients (11%) that was less than which founded in control group (23%). For genotypic frequency of heterozygous CT, which found to be higher in cancer patients (22%) compared to control group (19%), the frequency of homozygous genotype CC in cancer patients (17%), while in control group was (8%) and there were statistically significant differences between the patients’ group and control group.

For the allelic frequencies of C allele, there are statistically high significant differences between the cancer patients (n = 56) compared to control group (n = 35) where p = 0.0031. The obtained results of this study indicated, the codominant model of CC genotype of IFITM3 gene had high association with risk of hepatocellular carcinoma with odd ratio (OR) 2.70 with significant p = 0.041.

For the dominant model (CT + CC) of IFITM3 gene, the results showed that there is statistically high association with risk of hepatocellular carcinoma with odd ratio 3.02 and significant p = 0.01, the CC recessive model odd ratio = 2.70 and high association with risk of hepatocellular carcinoma.

[10] Reported that IFITM3 rs 12252-CC has been associated with the low differentiation and development of HCC. In HCC tissues and cells, IFITM3 was significantly expressed, and that expression was closely correlated with the prognosis and relapse of liver cancer.

The results of this study show that IFITM3 levels are significantly higher in HCC [11]. Indicated that IFITM3 was significantly overexpressed in HCC tissues and is related to a poor prognosis [12]. Showed that IFITM3 is overexpression in HCC, and is associated with tumor invasion and proliferation in HCC. IFITM3 CC genotype had higher expression than TT genotype.

According to [13] Limiting IFITM3 might prevent HCC cells from developing, spreading, invading to adjacent organs, and undergoing apoptosis. The prognosis for HCC patients with overexpression of IFITM3 was considered essential.

[14] Showed how IFITMs are involved in the growth of tumors. A poor prognosis has been related to the expression of the IFITM1, IFITM2, or IFITM3 proteins in a number of cancers, including colorectal, prostate, ovarian, lung, liver, breast, and astrocytomas. As a result, IFITMs have been recommended as a prognostic biomarker for a number of cancer types. Furthermore, in both human and mouse colorectal cancers, independent research has connected the expression of the IFITM3 gene to colon carcinogenesis, one of the most frequent causes of HCC.

## Conclusions

The study suggested that the CC genotype of IFITM3 rs 12252-CC polymorphisms might be associated with the risk of hepatocellular carcinoma and screening genotypes of IFITM3 for diagnosis and starting intervention of the disease is necessary.

## Electronic supplementary material

Below is the link to the electronic supplementary material.

Supplementary Material 1

## Data Availability

In the article or as supplemental information, all information essential to the study has been included.

## Abbreviations

HCC: Hepatocellular carcinoma.
IFITM3: Interferon-Induced Transmembrane protein-3.
ELISA: Enzyme-Linked Immune Sorbent Assay.
HWE: Hardy-Weinberg equilibrium.
SNP: Single nucleotide polymorphism
