# Supplementary material for: Interferon-induced transmembrane protein 3 in hepatocellular carcinoma patients
Source: BMC Cancer. 2023 Jun 23;23:584. doi: 10.1186/s12885-023-11071-2 (PMC10290370; doi:10.1186/s12885-023-11071-2)
Supplement: Supplementary file 1 — Supplementary Material 1 [file 12885_2023_11071_MOESM1_ESM.docx]

Links to NCBI databases

Gene

<https://www.ncbi.nlm.nih.gov/gene/?linkname=pmc_gene&from_uid=6336587>

Gene Nucleotide

<https://www.ncbi.nlm.nih.gov/gene/?linkname=pmc_gene_nucleotide&from_uid=6336587>

Geo profiles

<https://www.ncbi.nlm.nih.gov/geoprofiles/?linkname=pmc_geoprofiles&from_uid=6336587>

Med Gene

<https://www.ncbi.nlm.nih.gov/medgen/?linkname=pmc_medgen&from_uid=6336587>

Nucleotide

<https://www.ncbi.nlm.nih.gov/nucleotide/?linkname=pmc_nucleotide&from_uid=6336587>

Nucleotide

<https://www.ncbi.nlm.nih.gov/nuccore/?linkname=pmc_nuccore&from_uid=6336587>

SNP

<https://www.ncbi.nlm.nih.gov/snp/?linkname=pmc_snp&from_uid=6336587>

Taxonomy

<https://www.ncbi.nlm.nih.gov/taxonomy/?linkname=pmc_taxonomy&from_uid=6336587>

Protein

<https://www.ncbi.nlm.nih.gov/protein/148612842>
